# Internet-Based Universal Prevention for Students and Parents to Prevent Alcohol and Cannabis Use Among Adolescents: Protocol for the Randomized Controlled Trial of Climate Schools Plus 

**DOI:** 10.2196/10849

**Published:** 2018-08-17

**Authors:** Nicola Clare Newton, Cath Chapman, Tim Slade, Chloe Conroy, Louise Thornton, Katrina Elizabeth Champion, Lexine Stapinski, Ina Koning, Maree Teesson

**Affiliations:** ^1^ National Health and Medical Research Council Centre of Research Excellence in Mental Health and Substance Use National Drug and Alcohol Research Centre University of New South Wales Randwick Australia; ^2^ Feinberg School of Medicine Northwestern University Chicago, IL United States; ^3^ Faculty of Social and Behavioural Sciences Universiteit Utrecht Utrecht Netherlands

**Keywords:** alcohol, Australia, cannabis, parents, prevention, school, internet-based intervention

## Abstract

**Background:**

Early initiation of alcohol and cannabis use markedly increases the risk of harms associated with use, including the development of substance use and mental health disorders. To interrupt this trajectory, effective prevention during the adolescent period is critical. Despite evidence showing that parents can play a critical role in delaying substance use initiation, the majority of prevention programs focus on adolescents only. Accordingly, the *Climate Schools Plus* (CSP) program was developed to address this gap.

**Objective:**

This paper outlines the protocol for a cluster randomized controlled trial (RCT) of the CSP program, a novel internet-based program for parents and students to prevent adolescent substance use and related harms. The CSP program builds on the success of the *Climate Schools* student programs, with the addition of a newly developed parenting component, which allows parents to access the internet-based content to equip them with knowledge and skills to help prevent substance use in their adolescents.

**Methods:**

A cluster RCT is being conducted with year 8 students (aged 12-14 years) and their parents from 12 Australian secondary schools between 2018 and 2020. Using blocked randomization, schools are assigned to one of the two groups to receive either the CSP program (intervention) or health education as usual (control). The primary outcomes of the trial will be any student alcohol use (≥1 standard alcoholic drink/s) and any student drinking to excess (≥5 standard alcoholic drinks). Secondary outcomes will include alcohol- and cannabis-related knowledge, alcohol use-related harms, frequency of alcohol consumption, frequency of drinking to excess, student cannabis use, parents’ self-efficacy to stop their children using alcohol, parental supply of alcohol, and parent-adolescent communication. All students and their parents will complete assessments on three occasions—baseline and 12 and 24 months postbaseline. In addition, students and parents in the intervention group will be asked to complete program evaluations on two occasions—immediately following the year 8 program and immediately following the year 9 program.

**Results:**

Analyses will be conducted using multilevel, mixed-effects models within an intention-to-treat framework. It is expected that students in the intervention group will have less uptake and excessive use of alcohol compared with the students in the control group.

**Conclusions:**

This study will provide the first evaluation of a combined internet-based program for students and their parents to prevent alcohol and cannabis use.

**Trial Registration:**

Australian New Zealand Clinical Trials Registry ACTRN12618000153213; https://www.anzctr.org.au/Trial/Registration/TrialReview.aspx?id=374178 (Archived by WebCite at http://www.webcitation.org/71E0prqfQ)

**Registered Report Identifier:**

RR1-10.2196/10849

## Introduction

Alcohol and cannabis are the most commonly used licit and illicit drugs in Australia and are associated with substantial socioeconomic costs [1-4]. The initiation of substance use begins during adolescence, and early initiation markedly increases the risk of harms from use and subsequently developing substance use disorders [5]. To interrupt this trajectory, effective prevention during the adolescent period is critical. Traditional approaches to substance use prevention have focused on adolescents only; however, recent evidence suggests that expanding student interventions to include parenting components could markedly increase prevention effects [6-8]. This is because parents are key agents of adolescent socialization, especially in the initiation and development of substance use [9-13], and parenting interventions have been identified as critical components of effective substance use prevention programs [6,9,13,14]. Moreover, adolescent substance use is an area of substantial concern for parents, who generally want to be engaged in substance use harm prevention [15]. Parents also report that they actively seek information about parenting and adolescent substance use; however, most parents are not confident in their ability to stop their child from becoming drunk [16].

Despite the importance of including parents in prevention efforts, relatively few substance use prevention programs have involved both students and parents, and the programs developed have faced numerous challenges during their implementation (eg, high attrition rates, lack of engagement, and lack of sustainability [6,17]). Moreover, no substance use prevention programs that adopt an internet-based delivery approach have involved both parents and students, despite the potential for internet-based delivery to overcome some of the challenges encountered in the implementation and sustainability of prevention programs [6,17]. Therefore, the *Climate Schools Plus* (CSP) program was developed to address this gap and meet the need for a sustainable, evidenced-based student and parent prevention program. Building on the effective internet-based *Climate Schools* drug prevention programs for students [18-21], the CSP program combines the effective *Climate Schools: Alcohol and Cannabis* course for students aged 12-14 years [21-24] with a newly developed parent component [16]*.*

The course is an internet-based universal prevention program delivered to all students regardless of their level of risk and is based on a social influence approach to prevention [22]. The social influence approach has been found to be the most effective approach for school-based prevention programs in decreasing alcohol and cannabis use [22,25]; this approach involves delivering accurate information about substance use, placing substance use within a normative context (ie, most students their age are not using alcohol or cannabis), and developing students’ resistance skills (ie, their ability to identify sources of pressure to use substances and their ability to resist these pressures). The course covers these three components, using cartoon storylines to engage students and is delivered online to ensure high-implementation fidelity. The course consists of 12 internet-based lessons, which align with stage 5 of the Australian Health and Physical Education curriculum; it is designed to be implemented in early adolescence when youth are aged 12-14 years, before marked exposure to alcohol and cannabis occurs. The program has been trialed in two independent cluster randomized controlled trials (RCTs), which have demonstrated the effectiveness of the program in improving alcohol- and cannabis-related knowledge, reducing the uptake and harmful use of alcohol and the frequency of cannabis use up to 2 years following the intervention [18-21]. In addition, the program has been found to reduce psychological distress, moral disengagement (ie, the tendency to disengage from moral self-control and responsibility that ordinarily governs behavior, which has been associated with a range of antisocial behaviors, including heavy drug use and alcohol consumption in young people [26-30]), and truancy [22]. In light of recent evidence, which suggests that expanding universal interventions to include parenting components could markedly increase prevention effects [6], we developed the CSP program [16].

The parent component for the CSP program is based on the successful Dutch Prevention of Alcohol Use in Students (PAS) program [31-33] and was adapted for Australia in consultation with Australian parents and education and health experts [16]. The parent component of the CSP program is designed to be delivered entirely online, across the same school terms as the student *Climate Schools* program. The parent component targets modifiable parenting factors associated with adolescents’ alcohol and cannabis initiation and misuse [10,34], including rule-setting, parental supply, modeling, and monitoring. The parent component comprises introductory webinars, a rule-ranking component, internet-based modules, and internet-based parent summaries of the material covered in the student program. A complete description of the parent component can be found in a development paper published by the members of the research team [16].

Currently, we are seeking to evaluate the effectiveness of the CSP program in preventing alcohol and cannabis use and increasing parents’ self-efficacy to prevent their child from using these substances. Incorporating an internet-based parent component into an effective school-based student program has the potential to remarkably enhance prevention outcomes and reduce alcohol- and cannabis-related harms among adolescents.

**Figure 1:**
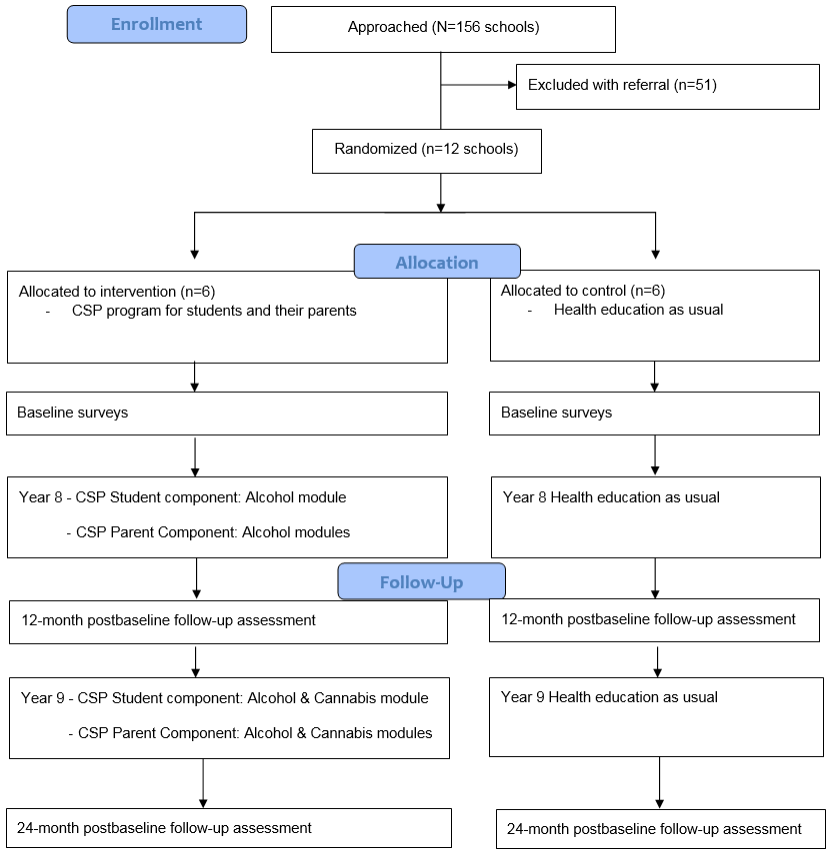
The *Climate Schools Plus* (CSP) intervention trial flow based on the Consolidated Standards of Reporting Trials (CONSORT) guidelines.

We will determine the effectiveness of the CSP intervention by running a cluster RCT in Australian Independent and Catholic secondary schools (ACTRN12618000153213). Cluster randomization will be used to avoid contamination of the control group with the intervention groups through student and staff communication. Schools will be randomly allocated to the CSP condition or the “control” condition. Students and parents in the CSP condition will be provided access to the *Climate Schools*: *Student* component and the *Climate Schools Parenting Program*, respectively, whereas students in the control condition will receive their health education classes as usual, which covers alcohol and other drug education topics. Students and parents in both groups will complete self-report surveys at baseline and 12 and 24 months postbaseline. Figure 1 depicts the study design.

## Methods

### Participants, Interventions, and Outcomes

#### Participants

##### Study Setting and Recruitment

This study is set in Independent and Catholic secondary schools in Australia. We obtained Human Research Ethics Committee (HREC) approval to approach Independent schools (HREC 17852) and Catholic schools in two dioceses in the Greater Sydney area (reference: 201731 and 060318) and invite them to participate. In October 2017, 156 schools were approached using a variety of methods, including advertisement during the Personal Development Health and Physical Education (PDHPE) Teachers’ Association Annual Conference, emails sent directly to schools or school principals, newsletter entries (ie, the Independent Secondary Schools Association and *Climate Schools* newsletters), posts on social media, and through follow-up phone calls. Parents will first be recruited to participate in the CSP study when their students bring home the parent permission form, along with a postcard containing information about the study and instructions on how to register. Then, they will also be recruited through an email sent from their school with information about the study, a link to a short, 45-second video introducing the aims of the study and instructions on how to register; this involves parents consenting for their adolescent’s and their own participation. Next, to access the content of the program, parents will need to register for an account through the CSP website. Students who have received parental permission to participate in the study will be approached to register and consent during their regular PDHPE classes and will be given the opportunity to send an email to their parents, reminding them to register for the program through an “Invite Your Parent” icon on the CSP website.

##### Sample Size

For cluster randomization, sample size calculations were based on sample size requirements developed by Heo and Leon [35] to detect the intervention by time interactions in longitudinal cluster randomized clinical trials. This trial is powered to detect differences in the overall student sample across three time-points. Five schools, with an average of 70 students per school, are required per intervention group; this would achieve an 80% power to detect a standardized, between-group mean difference of 0.2 (*P*=.05) in primary outcomes at the end of the trial, with 3 measurement occasions. An effect size of 0.2 is comparable with previous trials of combined student and parent programs (effect size range: 0.2-0.3) [36]. To account for school dropouts during the trial, which we expect to be approximately 15%, we aim to recruit at least 12 schools in total. Assuming that the majority (if not all) students in the year group participate in this study (approximately 70 on average; based on participation rates found in previous school-based trials of a similar nature [18-21,23,24,37]), it will give us a total of 700 students from 10 schools at the baseline to test the effect of the intervention.

##### Eligibility Criteria

Eligible participants are students attending participating schools and are enrolled in year 8 in 2018 and these students’ parents. These students will be 12-14 years of age at baseline and 14-16 years of age at the final assessment point. Furthermore, to be eligible to participate, students and parents must have at least intermittent internet access and basic proficiency in English.

##### Consent or Assent

After school principals agree to participate in the study, active consent will be sought individually from parents, students, and teachers. Parents will be asked to provide active consent for (1) their child’s participation in the study and (2) their own participation. Eligible students with parental consent will be directed to the participant information statement and consent form when registering on the CSP website, and all parents will similarly be directed to their participant information statements and consent forms when registering for the first time. Conversely, students and parents in the CSP group who do not consent to participate in the research trial will still be offered access to the content of the program; however, they will not be prompted to complete assessment surveys, and no data will be collected from those individuals.

#### Interventions

##### Active Intervention (Climate Schools Plus Group)

###### Climate Schools Plus: Student Component

**Figure 2:**
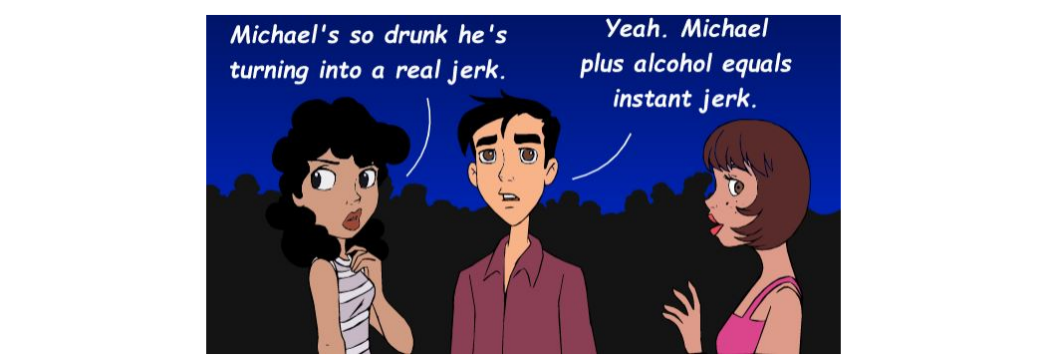
Screenshot of example student cartoon. Source: Netfront Pty Ltd.

The student component of the intervention consists of the effective and validated *Climate Schools: Alcohol* and modules [18-21,23,24,37], which involve 12, 40-minute lessons aimed at reducing alcohol and cannabis use and related harms. The first 6 lessons focus specifically on alcohol and are delivered in year 8, the remaining 6 lessons focus on alcohol and cannabis and are delivered 12 months later when the students are in year 9, prior to the development of harmful patterns of alcohol and cannabis use [38]. Therefore, the *Climate Schools* lessons are designed to avoid the development of harmful use by intervening when students are aged 12-14 years. The program will be completed at school, during regularly scheduled PDHPE classes, and to access the material, teachers, parents, and students are asked to create unique confidential log-in details on the CSP study website. The first part of each *Climate Schools* lesson is in the form of an internet-based cartoon storyline completed individually by students, which imparts information about alcohol (in years 8 and 9) and cannabis (in year 9; see Figure 2 for an example of the cartoon content). The second part of each lesson consists of optional class activities delivered by the teacher, such as role-plays and group discussions, which reinforce the information in the cartoons and allow communication among students. Teachers are provided access to an internet-based teacher’s manual, which contains lesson activities, implementation guidelines, links to the syllabus, and teacher summaries for each lesson.

###### Climate Schools Plus: Parent Component

**Figure 3:**
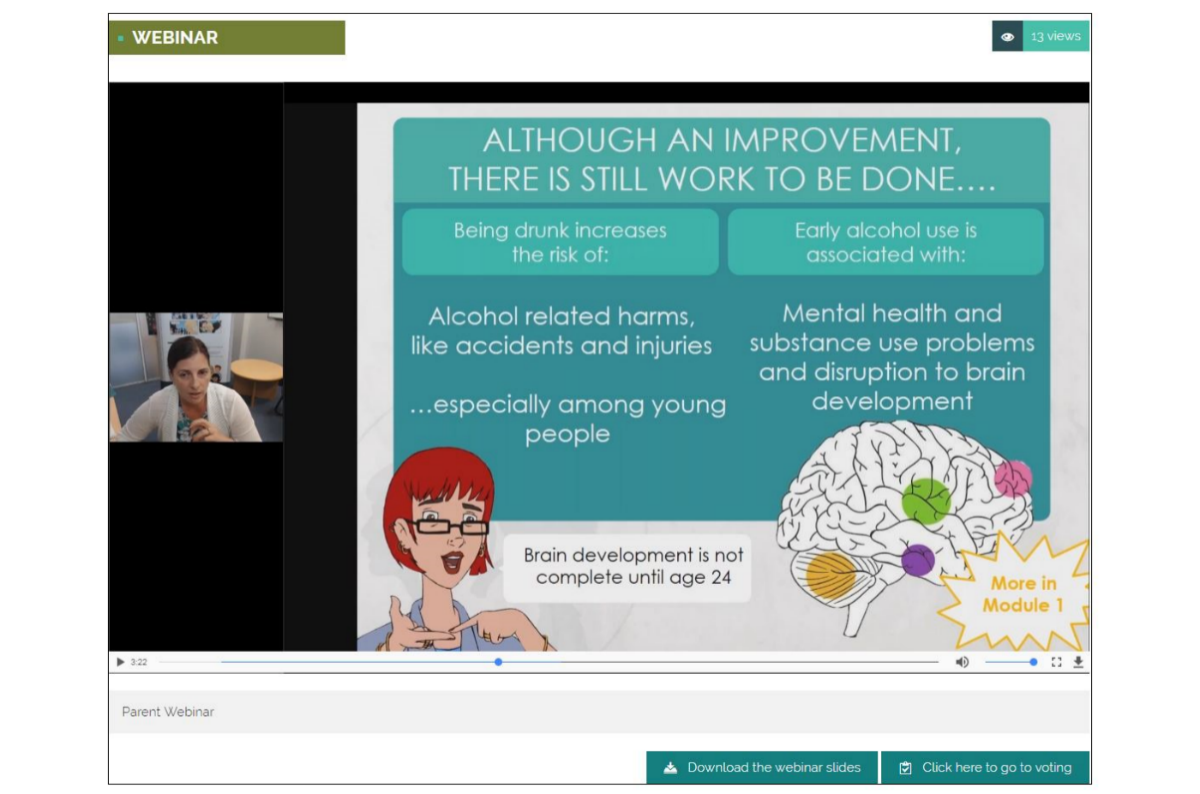
Screenshot of the year 8 webinar. Source: Netfront Pty Ltd.

**Figure 4:**
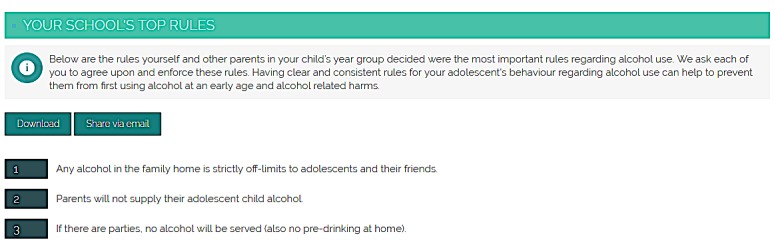
Screenshot of the rule-ranking component (when top 3 rules have been finalized). Source: Netfront Pty Ltd.

The parent component was developed in consultation with parents of Australian secondary school students and relevant experts through a large scoping survey, beta-testing, and pilot-testing of the developed program [16]. At the beginning of the study, parents will be sent an email from their school with information about the CSP program and instructions on how to register. Once parents have registered with their username, password, and unique school code, they will have access to 2 webinars (approximately 5 minutes each, at the beginning of years 8 and 9). These webinars are hosted by CC, who is a senior research fellow at the National Health and Medical Research Council (NHMRC) Centre of Research Excellence in Mental Health and Substance Use and also a member of the research team. The webinars provide overviews of alcohol and cannabis use in adolescents and related harms and highlight the role parents can play in preventing substance use in their child. During the webinar, parents will also be encouraged to engage in the “rule-ranking” component of the program, which allows parents from the same school to rank a series of rules related to alcohol use (eg, “Any alcohol in the family home is strictly off-limits to adolescents and their friends”); this component aims to facilitate a collective understanding of alcohol prevention and the role parental rule-setting plays in prevention by facilitating agreement among parents on a shared set of rules, which they can implement on their adolescents as a group. The top 3 rules will be published after 6 weeks, and parents will be encouraged to view and implement these rules with their adolescents over the school year (see Figures 3 and 4 for example screenshots of these components).

**Figure 5:**
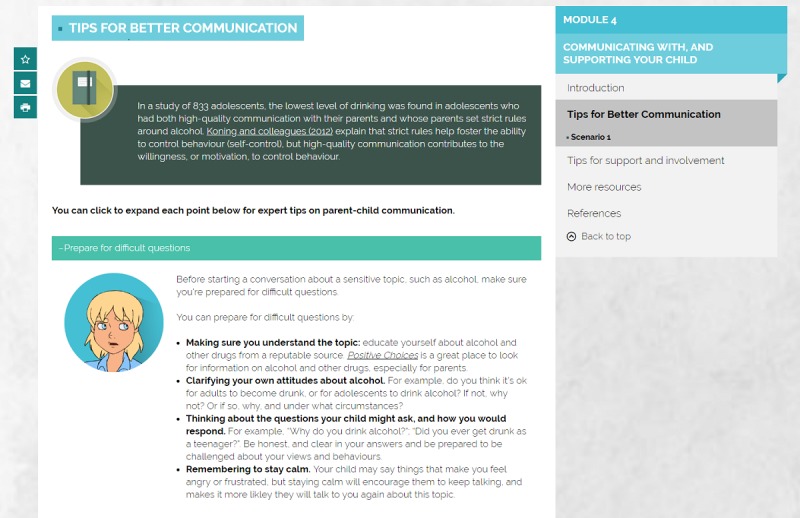
Screenshot of one of the parent modules (Parent Module 4, in the year 8 program). Source: Netfront Pty Ltd.

**Figure 6:**
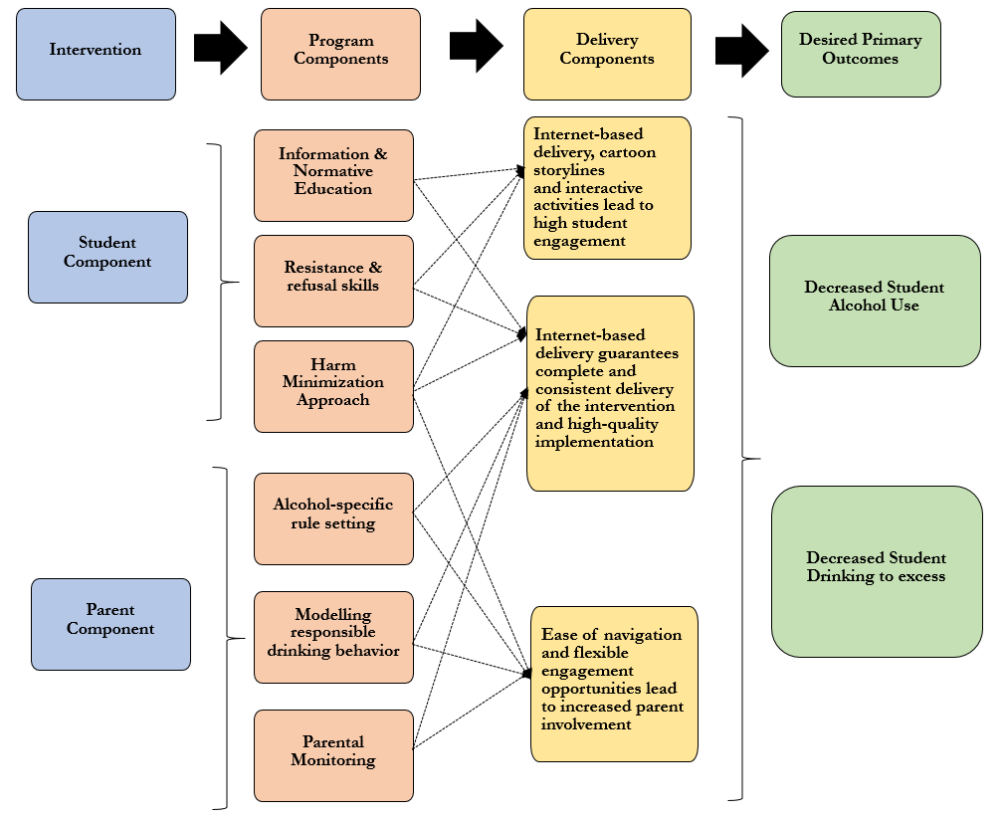
Theory of Change Logic Model for *Climate Schools Plus*.

Parents will also have access to 6 brief internet-based modules (under 10 minutes each, 4 in year 8 and 2 in year 9) covering a range of topics about alcohol and cannabis use, as well as parenting strategies and communication tips (Textbox 1; Figure 5). Parents can access the year 8 content anytime over the course of the study, whereas the year 9 content will be locked until the beginning of 2019. Parents will be sent occasional reminders through their school and the CSP website informing them when new content is available. Parents will also be emailed weekly summaries of the content covered in each of the *Climate Schools* student lessons. Figure 6 provides further information regarding the parent intervention and how it relates to the outcomes of the study.

##### Control Group

Students and their parents will follow the same registration and consent process in both the control group and the CSP group and will be asked to complete the baseline questionnaire. Then, students will receive their regular drug and alcohol education during their PDHPE classes (ie, they will cover topics such as drug use, health and well-being, sources of support, personal safety, exploring risk, etc, in accordance with the student outcomes defined by the Australian National Health and Physical Education syllabus) during the study. These students will be asked to complete the 12-month follow-up questionnaire in 2019 and the final follow-up questionnaire in 2020. Similarly, parents in the control group will be asked to complete the baseline questionnaire and 12- and 24-month follow-up questionnaires over the same period.

#### Participant Timeline

Students and parents in the CSP group will be invited to register for the CSP program online during the first year of the study (for the majority of schools, this will be during term 1 of 2018; however, for some, it will be term 2 or 3 of 2018). Students will complete their registration and baseline survey during their regularly scheduled PDHPE classes, and their teachers will facilitate their progression through the *Climate Schools: Alcohol* lessons. During the first week of the intervention, parents register for the program and are invited to view the webinar, participate in the interactive rule-ranking component, and explore the available modules. Approximately 8 weeks after registering for the program, parents and students will be prompted to complete an evaluation of the program content online, through the CSP website. Similarly, in 2019, students will complete the 12-month follow-up questionnaire, and their teacher will facilitate their progression through the lessons. Simultaneously, parents of these students will be invited to view the second webinar, the second round of rule-ranking, and invited to explore year 9 parent modules. Students and parents will also be asked to complete an evaluation of the year 9 program through the CSP website, as they did in the previous year. Finally, in 2020, parents and students will be asked to complete the 24-month follow-up questionnaire; a reminder email will be sent to nonresponding parents and students 1 week after they are invited to complete it and another reminder 1 week later. Table 1 summarizes the student and parent involvement in the study.

#### Ancillary or Posttrial Care

Upon conclusion of the trial, all schools in the control group will receive access to the same materials offered to the CSP group, free of charge.

**Overview of content in *Climate Schools Plus* parent modules.**

Module 1: Prevalence, patterns, and harms of adolescent alcohol use

Module 2: Parental attitudes and rule-setting

Module 3: Parental supply and use

Module 4: Communication and involvement

Module 5: Prevalence, patterns, and harms of adolescent cannabis use

Module 6: What parents can do to prevent adolescent cannabis use

#### Outcomes

We selected the following primary outcomes to reflect the overall target of the intervention (ie, to prevent alcohol use and related harms in adolescents). All primary outcomes will be measured at baseline and 12- (prior to the delivery of the year 9 intervention) and 24-month follow-up, with the primary endpoint being 24 months.

##### Primary Outcomes

###### Any Alcohol Use

Any alcohol use is defined as the consumption of at least 1 full serve or standard drink of alcohol (ie, any drink containing 10 grams of alcohol). To measure this outcome, students would be provided with a chart used to illustrate a standard drink (as used in the NHMRC Australian Guidelines [39]) and asked *“Have you ever had at least one standard alcoholic drink?”* and *“Have you had at least one standard alcoholic drink in the past 12 months?”* Responses to both questions are in the form of “Yes” versus “No.”

###### Any Drinking to Excess

Any drinking to excess is defined as the consumption of ≥5 standard drinks on a single occasion, in line with the NHMRC Australian Guidelines for risky drinking on a single occasion [39]. These alcohol use measures were used in the National Drug Strategy Household Survey [4], the Australian Secondary Students Alcohol and Drug Survey [38], and previous *Climate Schools* trials [18-21,23,24,37] and allows for comparisons between this sample and a large-scale representative group of Australians. Specifically, students would be asked *“Have you ever had 5 or more standard alcoholic drinks on one occasion?”* and *“Have you had 5 or more standard alcoholic drinks on one occasion in the past 12 months?”* Responses to both questions are in the form of “Yes” versus “No.”

##### Secondary Outcomes

All secondary outcomes will be assessed at baseline and 12- (prior to the delivery of the year 9 intervention) and 24-month follow-up, except for cannabis-related knowledge and patterns of cannabis use, which will only be measured at 12- and 24-month follow-up.

###### Alcohol-Related Harms

This outcome will be assessed using the 23-item Rutgers Alcohol Problem Index, which measures the consequences of alcohol use. Students would be asked to report the consequences of their alcohol use over the past 12 months, in which higher scores indicate greater harms [40].

###### Parental Self-Efficacy

Data regarding parental self-efficacy will be collected by a 3-item scale measuring parents’ confidence in their ability to prevent their adolescent from drinking alcohol, in which higher scores indicate a greater sense of self-efficacy [41]; this has been used and validated in the past PAS studies [42,43].

###### Parental Supply of Alcohol

This will be measured by a 2-question scale from the Australian Parental Supply of Alcohol Longitudinal Study (APSALS) [44] to examine the frequency and quantity of alcohol supplied by parents; higher scores indicate a higher frequency and quantity of alcohol supplied.

**Table 1:** The timeline of student and parent or guardian participation in the study.

Timeline		*Climate Schools Plus*		Control	
		Parent	Student	Parent	Student
**Term 1, 2018**					
	Assessment	Parent survey 1: baseline	Student survey 1: baseline	Parent survey 1: baseline	Student survey 1: baseline
	Intervention	Parent modules: alcohol	Year 8 student lessons: alcohol	—	—
	Evaluation	Evaluation of alcohol modules	Evaluation of alcohol lessons	—	—
**Term 1, 2019**					
	Assessment	Parent survey 2 (12-month follow-up)	Student survey 2 (12-month follow-up)	Parent survey 2 (12-month follow-up)	Student survey 2 (12-month follow-up)
	Intervention	Parent modules: alcohol and cannabis	Year 9 student lessons: alcohol and cannabis	—	—
	Evaluation	Evaluation of alcohol and cannabis modules	Evaluation of alcohol and cannabis lessons	—	—
**Term 1, 2020**					
	Assessment	Parent survey 3 (24-month follow-up)	Student survey 3 (24-month follow-up)	Parent survey 3 (24-month follow-up)	Student survey 3 (24-month follow-up)

###### Use of Cannabis and Other Drugs

Cannabis and other drug use (tobacco or cigarettes, amphetamines, ecstasy, hallucinogens, sedatives, inhalants, or “other”) will be measured by 5 questions adapted from the National Drug Strategy Household Survey [4] and the Australian Secondary Students Alcohol and Drug Survey (2014) [38], which have been used in previous *Climate Schools* trials to measure cannabis and other drug use [18-21,23,24,37]. For example, students would be asked *“Have you used cannabis in the past 12 months?,”* in which response options are “Yes” versus “No,” and *“How often have you used cannabis in the past 12 months?,”* in which response options range from “Never” to “More than once a day,” and higher scores indicate more frequent cannabis use. This allows for comparisons between this sample and a large-scale representative group of Australians.

###### Parent-Adolescent Communication

This outcome will be measured by a 6-question scale from the APSALS, examining parental knowledge and child disclosure of activities, friends, and whereabouts (eg, *“I usually know what my child is doing after school*,*”* with response options ranging from “Never” to “Always”; higher scores indicate a higher quality of communication). This outcome will also be measured by the 20-item Parent-Adolescent Communication Scale [45], which measures how cohesive and adaptable communication is between parents and children (eg, *“I can discuss my beliefs with my child without feeling restrained or embarrassed”* with response options ranging from “Strongly Disagree” to “Strongly Agree”).

###### Alcohol-Related Knowledge

Alcohol-related knowledge will be assessed using a 16-item “Knowledge of Alcohol” scale originally adapted from the School Health and Alcohol Harm Reduction Project (SHAHRP) questionnaire and used in previous trials of the *Climate Schools* programs [18-21,23,24,37,46]. Students and parents would be required to answer “True” or “False” to each item (eg, *“The recommended guidelines say that it is OK for adults to have up to 2 drinks on any one day”*), and a greater proportion of correct answers indicates greater alcohol knowledge.

###### Cannabis-Related Knowledge

Parents’ and students’ knowledge about cannabis will be assessed by 16 items of the “Knowledge about Cannabis” scale adapted from the SHAHRP questionnaire, as used in previous trials of the *Climate Schools* programs [18-21,23,24,37]. Students and parents would be required to answer “True” or “False” for each item, for example, *“Using cannabis can cause people to feel anxious, depressed (sad), paranoid (suspicious) and panicky,”* and a greater proportion of correct answers indicates greater cannabis knowledge.

###### Frequency of Alcohol Consumption

Students will be asked to report how frequently they consumed alcohol, in terms of standard drinks; this outcome will be measured by questions that were originally adapted from the SHAHRP “Patterns of Alcohol” index and have been used in previous *Climate Schools* trials [18-21,23,24,37,46]. Students would be asked questions such as*“How often did you have a standard alcoholic drink of any kind in the past 12 months?,”* with response options ranging from “Less than monthly” to “Daily or almost daily,” and higher scores indicating a higher frequency of alcohol consumption.

###### Frequency of Drinking to Excess

Students will be asked to report the frequency of drinking to excess (defined as having ≥5 standard drinks on a single occasion). These questions were originally adapted from the SHAHRP “Patterns of Alcohol” index and reflect those used in previous *Climate Schools* trials [18-21,23,24,37,46]. Students would be asked to report *“How often did you have 5 or more standard alcoholic drinks on one occasion in the past 12 months?”* with response options ranging from “Less than monthly” to “Daily or almost daily” and higher scores indicating a higher frequency of drinking to excess.

Additional measures include demographic information, such as gender, age, country of birth, truancy rates, and academic performance (to determine the baseline equivalence of groups), which will be assessed using questions that have been included in previous *Climate Schools* trials [18-21,23,24,37]. Students will also be asked to complete the Kessler 6 scale [47] to assess psychological distress in the past 30 days, and their quality of life will be measured by the Child Health Utility 9D scale [48]. Finally, students’ self-control will be measured using a 13-item scale developed by Tangney et al [49], which was used in the PAS program [32,33,42] and which includes questions such as *“I am good at resisting temptation*,*”* in which students would be asked to indicate how they typically are on a 5-point scale from “Not at all” to “Very Much.” These additional measures will be assessed at baseline and 12- and 24-month follow-up.

#### Assignment of Interventions

##### Allocation

Following school consent, schools have been randomly allocated to the CSP or control group by an external researcher; this process involved stratified random allocation, in which schools were divided into three mutually exclusive strata: (1) coeducational (mixed males and females) school, (2) single-sex or predominantly girls school, and (3) single-sex or predominantly boys school. The allocation was random within strata to achieve balance across intervention and control groups with respect to the number of males and females participating in the trial. Randomization was achieved using a randomization table created in StataSE, version 14, using the Stratarand procedure. The randomization sequence was computer-generated by an external biostatistician, who then informed the research team of which group each school had been allocated to.

##### Blinding (Masking)

It is not possible for the research team to remain blinded to the group allocation of schools during the study, given the nature of the intervention and the need to manage schools (particularly teachers as they progress through the program). However, the research team were not involved in the *allocation* of schools to the control or intervention groups, as this allocation was conducted externally, thereby removing the possibility of bias in the group allocation. Furthermore, the research team will not be in direct contact with the students or parents during the study (with the exception of students who are absent from school on the day of assessment; a list of absent students will be provided by the school teachers and these students will be contacted through email and invited to complete subsequent follow-up assessments by the research team). As such, no bias is likely to result from the research team’s knowledge of each school’s group allocation.

### Data Collection, Management, and Analysis

#### Data Collection

Students and parents will complete a self-assessment questionnaire online through the CSP website at baseline and 12- and 24-months postbaseline. The questionnaires contain scales used in previous *Climate Schools* trials, as well as specific parenting scales from the APSALS survey [44] and scales used to evaluate the effectiveness of the PAS program [42].

All measures are self-reported, as it has been found to be the most favored method of assessment for young people and has excellent discriminant [50] and predictive [51] validity [46,52]. Self-report has also previously been found to be a reliable method for assessing the frequency and quantity of adolescent alcohol use [53]. Furthermore, currently, no viable alternatives exist for data collection on alcohol use in an adolescent sample, as biological measures would not be appropriate in a sample at the early stages of alcohol use initiation [54].

##### Intervention Fidelity

Internet-based tracking will be used to monitor the extent to which students and parents engage with the intervention (ie, tracking the number of webinar views, the number of parents who ranked the rules, the number of parents who completed each module, the number of students who completed each lesson, etc). While teachers in the intervention group will be asked to complete a logbook as a record of how they delivered the intervention, teachers in the control group will complete a logbook detailing the type of drug and alcohol education they offered to their students over the same period.

##### Program Evaluation

Students, parents, and teachers in the CSP group will also be asked to complete two additional questionnaires, asking them to evaluate the intervention content that they received (ie, they will be asked how acceptable, appropriate, and enjoyable they found the program content). Teachers will be asked to indicate the likelihood that they would recommend the program to others, whereas students and parents will be asked to rate the likelihood that they would use the information and skills they learned in their own lives. Students and parents will be invited to complete these evaluation questionnaires once in 2018 after completing the *Alcohol* program and again in 2019 after completing the *Alcohol and Cannabis* program.

#### Retention

To facilitate the retention of students and parents in the trial, frequent communication will be maintained through email prompts generated from the CSP website, which reiterates the requirements of the study. Participants will also be offered reimbursement after completing each study questionnaire; specifically, students will be entered into a draw to win one of three Aus $500 JB Hi-Fi or Apple store vouchers at each assessment occasion, and parents will enter the draw to win one of three Aus $500 Westfield or Coles gift cards. Moreover, teachers will receive a one-off reimbursement of Aus $50 for the extra administration involved during the trial. When completing the baseline questionnaire at the beginning of the study, students and parents will be asked to enter their contact details (ie, name, address, home number, mobile number, and secondary email), which will be used solely for contacting students who are absent from school on the day of a questionnaire (absent students will be identified by their teacher, who will inform the research team of the absent students) and inviting them to complete subsequent follow-up assessments. Any data collected from students and parents who have consented but discontinue the study will be used in the analysis, in accordance with the intention-to-treat principle. The chosen statistical analysis techniques consider missing data resulting from participant discontinuation.

#### Statistical Analysis

In this study, primary and secondary outcomes will be analyzed in longitudinal analyses using multilevel mixed-effects regression models. The effects of highest interest are intervention × time interactions that reflect differences between intervention groups in the growth of each outcome over time. The multilevel modeling can account for the expected correlations between different observations of the same individual and between individuals in the same school [55], which would otherwise violate assumptions of independence in traditional regression models. Therefore, models used in these analyses will incorporate both random intercepts and slopes for time at the individual level and random intercepts at the school level. Mixed-effects regression approaches accommodate missing data through the maximum likelihood estimation, an approach that is superior to alternative missing data strategies such as pairwise deletion [56]. Maximum likelihood methods produce unbiased estimates when missing data are assumed to be either missing completely at random or missing at random [57].

Mixed-effects logistic regression with a logit link function will be applied when analyzing binary outcomes. A range of potential fixed effects and random effects structures will be compared using likelihood ratio tests and model fit statistics, such as the Akaike information criterion, to determine the best fitting model for each outcome. For all outcomes, between-condition effect sizes (eg, Cohen *d*) and odds ratios will be calculated along with their corresponding 95% CIs, to provide interpretable estimates of the intervention effects. All analyses will be conducted on an intention-to-treat basis, retaining and analyzing all students and parents in the groups they were originally allocated.

#### Planned Comparisons

The primary aim of this study is to evaluate the efficacy of the CSP program in comparison with the standard health education received by the control group. Therefore, planned comparisons for each outcome will compare students and parents in the CSP condition with students and parents in the control condition at baseline and 12- and 24-month follow-up.

#### Monitoring

This study will be overseen by an external biostatistician, and any adverse events will be reported to the University of New South Wales Sydney HREC to maintain the integrity of the study including the data collected, trial progress, and ethical compliance. However, given that the intervention reflects normative alcohol and cannabis education provided as part of the PDHPE curriculum, no serious adverse events are anticipated to occur during the study; therefore, a formal steering committee is not required.

### Ethics and Dissemination

#### Research Ethics Approval

Ethics approval was obtained by the University of New South Wales Sydney HREC (HC17852), the Sydney Catholic Education Office (Ref: 201731), and Catholic Education Parramatta (Ref: 060318).

#### Confidentiality

Confidentiality of the collected information will be strictly maintained, and participants’ data will remain anonymous. To access internet-based questionnaires and materials, students and parents will be required to register on the CSP website and once logged in, all data collected will be automatically deidentified, and the database will generate a unique ID code for each participant and the individual’s data files across sessions will be linked with this unique ID code. All data collected will be in a computerized format and stored in password-protected files on university servers, accessible only to the research staff and stored separately to questionnaire data. These procedures mirror those used in previous and existing school-based prevention trials conducted by the research team (eg, The CAP Study, HREC 11274 [50]).

#### Dissemination Policy

The results of this study will be presented to academic peers at conferences and published in health and education peer-reviewed journals. The feedback will be provided to participating schools in the form of a deidentified report of the study’s findings. This report will also be available to students and their parents at the end of the study. When publishing results of this study, no information will be published on the basis of individual cases, and all published data will reflect group data.

### Trial Funding

This study is supported by funding from the Australian Government Department of Health and a Society for Mental Health Research Early Career Research Award to NCN. This study was also funded by the NHMRC through the NHMRC Centre of Research Excellence (APP1041129).

## Results

This study is funded by the Australian Government Department of Health from 2016 to 2020 and by Society for Mental Health Research Early Career Research from 2015 to 2017. Enrollment of schools began in January 2018, with 8 out of 12 schools enrolled at the time of submission (enrollment is expected to be complete by October 2018). Baseline assessments are currently underway, and the first results are expected to be submitted for publication in 2019.

## Discussion

### Trialing Climate Schools Plus

This paper describes the design and protocol of the CSP study, the first international trial of an integrated internet-based intervention for students and parents to prevent alcohol and cannabis use. The effectiveness of the CSP program will be assessed through a cluster RCT, relative to health education as usual at 12- and 24-months postbaseline. Contamination will be avoided by the use of a cluster RCT design, in which each school forms its own cluster and is allocated to either the intervention or control group, preventing individuals at the same school being allocated to separate conditions and thus preventing contamination between staff and students at each school. We aim to ascertain whether a combined internet-based approach to drug prevention can be effective in preventing alcohol use among adolescents and improving parents’ self-efficacy to prevent their children from using substances.

### Strengths and Limitations

To date, approaches to substance use prevention have traditionally focused on adolescents themselves, despite evidence suggesting that parents play a critical role in substance use initiation [10-13]. The CSP study addresses the need for an integrated program for both students and their parents that is potentially sustainable through internet-based facilitation. The CSP program is built on a decade of sound research, which has shown that the internet-based *Climate Schools* programs for students are effective in preventing substance use. The expansion of this model to involve parents has the potential to improve prevention effects and provides a sustainable and scalable model for both students and parents.

The CSP program utilizes cartoon storylines to engage students and maintain their interest and internet-based technology to engage parents and improve ease of access to substance use prevention information, based on a successful evidence-based intervention [32,33]. Importantly, the internet-based nature of the CSP parent program (namely the on-demand webinars, modules, and parent summaries), provides parents with the flexibility to access the program material remotely at a time and location of their choice, which has the potential to improve the uptake of the program. Nonetheless, engagement and adherence are common challenges faced in trials of internet-based interventions [58,59]; therefore, this study uses a number of strategies to increase parent engagement (including frequent email communication, an “Invite Your Parent” icon displayed on students’ homepage, facilitation of a parent-generated “shared” set of school-specific rules among parents, etc). All of these engagement strategies are sustainable if the program were to be delivered outside of a trial setting. Importantly, the detailed measurement of internet-based engagement and interaction with the program will provide important information about the feasibility of engaging parents in internet-based substance use prevention trials in the future.

A potential limitation of this study is the use of self-report, which could be subject to the social desirability bias. However, previous research has demonstrated that self-report measures of substance use among adolescents have yielded excellent discriminant [50] and predictive [51] validity [46,52] and have been found to be a reliable method of assessing the frequency and quantity of adolescent alcohol use [53]. In addition, researchers will use strategies to maximize the accuracy of self-report, which have been successful in previous school-based trials [18-21,23,24,37,46]; these strategies include blind administration of any assessments within schools and a strong emphasis on anonymity and confidentiality.

Attrition is another potential limitation to this study, which could result from students not being present on the day of assessments or not providing correct or complete contact details to allow the research team to link their responses over time. However, missing data are likely to be at random, and the chosen data analytic techniques (mixed-effects regression modeling) use all available data, thus reducing the bias brought about by participant attrition.

The school sample used in this study (ie, Independent and Catholic school types) may limit the generalizability of the findings to the broader population. However, we do not expect this factor to markedly impact the outcomes of the trial, as previous research has found that the consumption and frequency of cannabis use within independent (nongovernment) schools was comparable to the larger population of young people of the same age [18,60], suggesting that the consumption and frequency of substance use is comparable between government and nongovernment school types. Furthermore, the research team aimed to recruit a range of schools from various geographic regions for the study and used a stratified randomization scheme when allocating schools to conditions to improve the generalizability of the findings.

An additional limitation of this study involves the need to obtain active consent from participants, as this might introduce selection bias. We aim to overcome this risk of selection bias with a robust study design. As this is an RCT, both participants in the intervention condition and control condition will volunteer to participate; therefore, the impact of volunteering is likely to be spread evenly across the two conditions. Although active or voluntary consent procedures can introduce selection bias [61], previous studies have found no differences in alcohol or illicit drug use among students with passive or active consent [62]. Moreover, we aim to minimize the chance of selection bias by offering support for participants, to ensure their understanding of the consent form and what is required if they participate in the study (ie, consent forms will be offered in both electronic and hard-copy forms, and if required, the form can be translated into the preferred language of the participants). Ideally, passive consent procedures would be used to maximize the number of participants taking part in the trial; however, ethical restrictions meant that we were required to use active consent procedures in this study. As such, future research would benefit from the use of passive consent procedures to reduce the impact of selection bias on the outcomes in the study.

### Conclusions

The CSP program was developed to address an unmet need for an integrated, internet-based program for students and parents to prevent alcohol and cannabis use. The CSP program fits within the school PDHPE curriculum and overcomes barriers to the implementation through online delivery, making it scalable to meet the needs of students and parents across Australia. If proven to be effective, this comprehensive program could be implemented widely, as part of a national strategy to significantly reduce the burden of disease, social costs, and disability associated with early substance use in adolescents.

## Abbreviations

APSALS: Australian Parental Supply of Alcohol Longitudinal Study
CSP: *Climate Schools Plus*
HREC: Human Research Ethics Committee
NHMRC: National Health and Medical Research Council
PAS: Prevention of Alcohol Use in Students
PDHPE: Personal Development Health and Physical Education
RCT: randomized controlled trial
SHAHRP: School Health and Alcohol Harm Reduction Project

## Appendix

Multimedia Appendix 1 Peer-reviewer report – Society for Mental Health Research.

## References

[1] Degenhardt L, Stockings E, Patton G, Hall WD, Lynskey M (2016). The increasing global health priority of substance use in young people. Lancet Psychiatry.

[2] Ciobanu LG, Ferrari AJ, Erskine HE, Santomauro DF, Charlson FJ, Leung J, Amare AT, Olagunju AT, Whiteford HA, Baune BT (2018). The prevalence and burden of mental and substance use disorders in Australia: Findings from the Global Burden of Disease Study 2015. Aust N Z J Psychiatry.

[3] Roche A, Pidd K, Kostadinov V (2016). Alcohol- and drug-related absenteeism: a costly problem. Aust N Z J Public Health.

[4] Australian Institute of Health and Welfare (2017). National Drug Strategy Household Survey 2016: detailed findings, in Drug Statistics series no. 31. PHE 214.

[5] Hall WD, Patton G, Stockings E, Weier M, Lynskey M, Morley KI, Degenhardt L (2016). Why young people's substance use matters for global health. Lancet Psychiatry.

[6] Newton NC, Champion KE, Slade T, Chapman C, Stapinski L, Koning I, Tonks Z, Teesson M (2017). A systematic review of combined student- and parent-based programs to prevent alcohol and other drug use among adolescents. Drug Alcohol Rev.

[7] Smit E, Verdurmen J, Monshouwer K, Smit F (2008). Family interventions and their effect on adolescent alcohol use in general populations; a meta-analysis of randomized controlled trials. Drug Alcohol Depend.

[8] Vermeulen-Smit E, Verdurmen JEE, Engels RCME (2015). The Effectiveness of Family Interventions in Preventing Adolescent Illicit Drug Use: A Systematic Review and Meta-analysis of Randomized Controlled Trials. Clin Child Fam Psychol Rev.

[9] Özdemir M, Koutakis N (2016). Does promoting parents' negative attitudes to underage drinking reduce adolescents' drinking? The mediating process and moderators of the effects of the Örebro Prevention Programme. Addiction.

[10] Yap MBH, Cheong TWK, Zaravinos-Tsakos F, Lubman DI, Jorm AF (2017). Modifiable parenting factors associated with adolescent alcohol misuse: a systematic review and meta-analysis of longitudinal studies. Addiction.

[11] Koning IM, van DERJJM, Verdurmen JEE, Engels RCME, Vollebergh WAM (2012). Developmental alcohol-specific parenting profiles in adolescence and their relationships with adolescents' alcohol use. J Youth Adolesc.

[12] Koning IM, van DERJJM, Engels RCME, Verdurmen JEE, Vollebergh WAM (2011). Why target early adolescents and parents in alcohol prevention? The mediating effects of self-control, rules and attitudes about alcohol use. Addiction.

[13] Garcia-Huidobro D, Doty JL, Davis L, Borowsky IW, Allen ML (2018). For Whom Do Parenting Interventions to Prevent Adolescent Substance Use Work?. Prev Sci.

[14] Onrust SA, Otten R, Lammers J, Smit F (2016). School-based programmes to reduce and prevent substance use in different age groups: What works for whom? Systematic review and meta-regression analysis. Clin Psychol Rev.

[15] Koning IM, van den Eijnden RJ, Glatz T, Vollebergh WA (2013). Don’t Worry! Parental Worries, Alcohol-Specific Parenting and Adolescents’ Drinking. Cogn Ther Res.

[16] Thornton LK, Chapman C, Leidl D, Conroy C, Teesson M, Slade T, Koning I, Champion K, Stapinski L, Newton N (2018). Climate schools plus: An online, combined student and parent, universal drug prevention program. Internet Interventions.

[17] Cairns G, Purves R, McKell J (2014). Combining school and family alcohol education: a systematic review of the evidence. Health Education.

[18] Newton NC, Andrews G, Teesson M, Vogl LE (2009). Delivering prevention for alcohol and cannabis using the Internet: a cluster randomised controlled trial. Prev Med.

[19] Newton NC, Teesson M, Vogl LE, Andrews G (2010). Internet-based prevention for alcohol and cannabis use: final results of the Climate Schools course. Addiction.

[20] Champion KE, Newton NC, Stapinski L, Slade T, Barrett EL, Teesson M (2016). A cross-validation trial of an Internet-based prevention program for alcohol and cannabis: Preliminary results from a cluster randomised controlled trial. Aust N Z J Psychiatry.

[21] Teesson M, Newton NC, Slade T, Carragher N, Barrett EL, Champion KE, Kelly EV, Nair NK, Stapinski LA, Conrod PJ (2017). Combined universal and selective prevention for adolescent alcohol use: a cluster randomized controlled trial. Psychol Med.

[22] Newton NC, Vogl L, Teesson M, Andrews G (2011). Developing the climate schools: Alcohol and Cannabis Module: a harm-minimization, universal drug prevention program facilitated by the internet. Subst Use Misuse.

[23] Newton NC, Andrews G, Champion KE, Teesson M (2014). Universal Internet-based prevention for alcohol and cannabis use reduces truancy, psychological distress and moral disengagement: a cluster randomised controlled trial. Prev Med.

[24] Vogl L, Teesson M, Andrews G, Bird K, Steadman B, Dillon P (2009). A computerized harm minimization prevention program for alcohol misuse and related harms: randomized controlled trial. Addiction.

[25] Gorman DM (2003). Alcohol & drug abuse: the best of practices, the worst of practices: the making of science-based primary prevention programs. Psychiatr Serv.

[26] Bandura A, Caprara GV, Barbaranelli C, Pastorelli C, Regalia C (2001). Sociocognitive self-regulatory mechanisms governing transgressive behavior. J Pers Soc Psychol.

[27] Pelton J, Gound M, Forehand R, Brody G (2004). The Moral Disengagement Scale: Extension with an American Minority Sample. Journal of Psychopathology and Behavioral Assessment.

[28] Passini S (2012). The delinquency-drug relationship: the influence of social reputation and moral disengagement. Addict Behav.

[29] Barnes GM, Welte JW, Hoffman JH, Dintcheff BA (1999). Gambling and alcohol use among youth: influences of demographic, socialization, and individual factors. Addict Behav.

[30] Newton NC, Havard A, Teesson M (2011). The association between moral disengagement, psychological distress, resistive self-regulatory efficacy and alcohol and cannabis use among adolescents in Sydney, Australia. Addiction Research & Theory.

[31] Koning IM, Vollebergh WAM, Smit F, Verdurmen JEE, Van DERJJM, Ter BTFM, Stattin H, Engels RCME (2009). Preventing heavy alcohol use in adolescents (PAS): cluster randomized trial of a parent and student intervention offered separately and simultaneously. Addiction.

[32] Koning IM, van DERJ, Verdurmen JE, Engels RC, Vollebergh WA (2011). Long-term effects of a parent and student intervention on alcohol use in adolescents: a cluster randomized controlled trial. Am J Prev Med.

[33] Koning IM, van DERJJM, Verdurmen JEE, Engels RCME, Vollebergh WAM (2013). A cluster randomized trial on the effects of a parent and student intervention on alcohol use in adolescents four years after baseline; no evidence of catching-up behavior. Addict Behav.

[34] Dorius CJ, Bahr SJ, Hoffmann JP, Harmon EL (2004). Parenting practices as moderators of the relationship between peers and adolescent marijuana use. Journal of Marriage and Family.

[35] Heo M, Leon AC (2009). Sample size requirements to detect an intervention by time interaction in longitudinal cluster randomized clinical trials. Stat Med.

[36] Newton NC, Conrod PJ, Slade T, Carragher N, Champion KE, Barrett EL, Kelly EV, Nair NK, Stapinski L, Teesson M (2016). The long-term effectiveness of a selective, personality-targeted prevention program in reducing alcohol use and related harms: a cluster randomized controlled trial. J Child Psychol Psychiatry.

[37] Vogl LE, Newton NC, Champion KE, Teesson M (2014). A universal harm-minimisation approach to preventing psychostimulant and cannabis use in adolescents: a cluster randomised controlled trial. Subst Abuse Treat Prev Policy.

[38] Drug Strategy Branch (2015). Australia Secondary School students' use of tobacco alcohol, and over-the-counter and illicit substances in 2014.

[39] (2009). National Health and Medical Research Council.

[40] Van Der Vorst H, Engels RCME, Meeus W, Deković M, Van Leeuwe J (2005). The role of alcohol-specific socialization in adolescents' drinking behaviour. Addiction.

[41] Koning IM, Maric M, MacKinnon D, Vollebergh WAM (2015). Effects of a combined parent-student alcohol prevention program on intermediate factors and adolescents' drinking behavior: A sequential mediation model. J Consult Clin Psychol.

[42] Glatz T, Koning IM (2016). The Outcomes of an Alcohol Prevention Program on Parents' Rule Setting and Self-efficacy: a Bidirectional Model. Prev Sci.

[43] White HR, Labouvie EW (1989). Towards the assessment of adolescent problem drinking. J Stud Alcohol.

[44] Aiken A, Wadolowski M, Bruno R, Najman J, Kypri K, Slade T, Hutchinson D, McBride N, Mattick RP (2017). Cohort Profile: The Australian Parental Supply of Alcohol Longitudinal Study (APSALS). Int J Epidemiol.

[45] Barnes HL, Olson DH (1985). Parent-Adolescent Communication and the Circumplex Model. Child Development.

[46] Conrod PJ, Castellanos N, Mackie C (2008). Personality-targeted interventions delay the growth of adolescent drinking and binge drinking. J Child Psychol Psychiatry.

[47] Kessler RC, Andrews G, Colpe LJ, Hiripi E, Mroczek DK, Normand SLT, Walters EE, Zaslavsky AM (2002). Short screening scales to monitor population prevalences and trends in non-specific psychological distress. Psychol Med.

[48] Stevens KJ (2010). Working with children to develop dimensions for a preference-based, generic, pediatric, health-related quality-of-life measure. Qual Health Res.

[49] Tangney JP, Baumeister RF, Boone AL (2004). High self-control predicts good adjustment, less pathology, better grades, and interpersonal success. J Pers.

[50] Newton NC, Teesson M, Barrett EL, Slade T, Conrod PJ (2012). The CAP study, evaluation of integrated universal and selective prevention strategies for youth alcohol misuse: study protocol of a cluster randomized controlled trial. BMC Psychiatry.

[51] Crowley TJ, Mikulich SK, Ehlers KM, Whitmore EA, MacDonald MJ (2001). Validity of structured clinical evaluations in adolescents with conduct and substance problems. J Am Acad Child Adolesc Psychiatry.

[52] Conrod PJ, Castellanos-Ryan N, Strang J (2010). Brief, personality-targeted coping skills interventions and survival as a non-drug user over a 2-year period during adolescence. Arch Gen Psychiatry.

[53] Koning IM, Harakeh Z, Engels RCME, Vollebergh WAM (2010). A comparison of self-reported alcohol use measures by early adolescents: Questionnaires versus diary. Journal of Substance Use.

[54] Carroll KM (1995). Methodological issues and problems in the assessment of substance use. Psychological Assessment.

[55] Fitzmaurice GM, Laird NM, Ware JH (2012). Applied Longitudinal Analysis.

[56] Schafer JL, Graham JW (2002). Missing data: our view of the state of the art. Psychol Methods.

[57] Hox JJ (2010). Multilevel analysis techniques and applications. Multilevel analysis techniques and applications.

[58] White A, Kavanagh D, Stallman H, Klein B, Kay-Lambkin F, Proudfoot J, Drennan J, Connor J, Baker A, Hines E, Young R (2010). Online alcohol interventions: a systematic review. J Med Internet Res.

[59] Cavanagh K, Strauss C, Cicconi F, Griffiths N, Wyper A, Jones F (2013). A randomised controlled trial of a brief online mindfulness-based intervention. Behav Res Ther.

[60] Australian Institute of Health and Welfare (2008). 2007 National Drug Strategy Household Survey: First Results.

[61] Shaw T, Cross D, Thomas LT, Zubrick SR (2014). Bias in student survey findings from active parental consent procedures. Br Educ Res J.

[62] Anderman C, Cheadle A, Curry S, Diehr P, Shultz L, Wagner E (2016). Selection Bias Related To Parental Consent in School-Based Survey Research. Eval Rev.

