# Supplementary material for: Internet-Based Universal Prevention for Students and Parents to Prevent Alcohol and Cannabis Use Among Adolescents: Protocol for the Randomized Controlled Trial of Climate Schools Plus
Source: JMIR Res Protoc. 2018 Aug 17;7(8):e10849. doi: 10.2196/10849 (PMC6119212; doi:10.2196/10849)
Supplement: Multimedia Appendix 1 [file resprot_v7i8e10849_app1.pdf]

society for mentalhealthresearch

Friday 15 May 2015

Nicole Newton
NHMRC Centre of Research Excellence in Mental Health and Substance Use
National Drug and Alcohol Research Centre, UNSW
nickien@unsw.edu.au

Dear Nicole,

Application title: An innovative response to improving the prevention of substance use and mental health problems among young Australians.

Thank you for your application to the inaugural Society for Mental Health Research Early Career Research Fellowship Scheme. We received 49 applications to this scheme in 2015. From this pool, 13 fellowships are being awarded to outstanding early career research fellows around Australia.

Our expert reviewers, drawn from our top ranking mental health research institutions across Australia made special comment on the high quality, competitiveness and calibre of all applications received. We are pleased to advise that your application was ranked among the top 13 fellowships applications received, and your application for an SMHR ECR Fellowship was successful.

Documentation to formalise your acceptance of the offer of an SMHR Early Career Fellowship will be provided to you and your Institution in the coming weeks. Your SMHR Early Career Fellowship will be of 12 months' duration. This award is to the value of $100,000, and should be divided between a contribution to the cost of your salary costs (up to $75,000 in total), and an allocation for project support as specified in your application ($25,000). All other costs associated with your salary are to be provided by your Institution.

For now, we require formal acceptance of your SMHR Early Career Fellowship Award from you, which should also contain the name and contact details of the person within your Institution's Research Office through which we can negotiate your appointment and transfer of funds. Please provide this information via email to smhr@smhr.org.au.

Thank you for the time and effort you put into this application, and for your obvious commitment to mental health research in Australia. Congratulations on your successful application, and we wish you every success with this Award.

Kind Regards,

S M Cotton

Associate Professor Susan Cotton
Convenor
Early Career Fellowship Review Panel
Society for Mental Health Research

Patrick D. McGorry

Professor Patrick McGorry
President
Society for Mental Health Research
